# Evaluation of the SwedeAmp database: Focus on coverage and amputation level rates

**DOI:** 10.33137/cpoj.v7i2.44089

**Published:** 2024-11-19

**Authors:** A.G Johannesson, R Scheving, k.L Westlund, T Fridriksson

**Affiliations:** 1 Össur Clinics EMEA, Stockholm, Sweden.; 2 Össur Iceland Ehf., R&D, Medical Office, Reykjavik, Iceland.

**Keywords:** Amputation, Rehabilitation, Lower Limb Amputation, SwedeAmp, Amputation Rates, Sweden, Transfemoral, Transtibial, Knee Disarticulation

## Abstract

**BACKGROUND:**

The National Board of Health and Welfare manages several national registers in Sweden. This includes the Swedish National Inpatient Register (IPR), covering all surgical operations, and SwedeAmp, focusing on outcomes after lower limb amputations (LLA). However, coverage rates of amputation levels between these registers have not been externally analyzed.

**OBJECTIVE:**

To compare SwedeAmp's coverage with IPR for LLA cases and to assess SwedeAmp's accuracy in capturing LLA data. The goal of this study was also to identify potential discrepancies and establish benchmarks for common amputation levels.

**METHODOLOGY:**

Data from both registers, covering the years 2018 to 2023, were compared regarding the amputation levels and patient demographics. The coverage rate of the SwedeAmp register was calculated using SwedeAmp data as the numerator and IPR data as the denominator.

**FINDINGS:**

The IPR registry recorded 10,788 LLAs across 21 regions (67 hospitals). The SwedeAmp documented 5,246 LLAs covering 17 regions (36 hospitals), leaving 5,542 amputations unaccounted for, mainly due to regions or hospitals not participating in the SwedeAmp registry and lower registration rates in some areas. Key findings include:

- Achieving full coverage in SwedeAmp (17 regions) would require registering 9,305 LLAs.
- Both men and women over 85 years were significantly underrepresented.
- Thirteen regions in SwedeAmp obtained more than 40% coverage rate.
- 5 regions had more than 50% rate of above-knee amputations (range: 50.9% – 68.2%).
- 8 regions reporting more than 50% rate of below-knee amputations (range: 53.1% – 88.9%).
- Among the 67 hospitals performing LLAs, 36 reported to SwedeAmp. Six of these hospitals performed fewer than 10 LLAs over a six-year period.

**CONCLUSION:**

SwedeAmp captured 48.6% of initial LLAs in Sweden, highlighting the need for improved data completeness in LLA records, especially as only 13 regions achieved over 40% Coverage. For hospitals performing regular amputation, the proposed benchmark-coverage of ≥60%, with ≤36.3% for transfemoral amputation (TF), ≤8.4% for knee disarticulations (KD), and ≥55.3% for transtibial amputations (TT) – could serve as a target to enhance consistency and accuracy in reporting. Expanding coverage can improve the register's utility in tracking outcomes, setting national standards, aiding research, and supporting clinical decision-making.

## INTRODUCTION

National patient registers collect data on diseases and treatments within specialized care, covering all inpatient admissions and outpatient doctor visits in these setting. A register can be used to monitor long-term health trends in the population, improve the prevention and treatment of diseases, contribute to the development of health care, and monitor the quality of health care services.^1^ In lower limb amputation (LLA), this can be the only practical option to evaluate the selection of amputation level due to ethical considerations, cost, and practicality. In developed countries, elderly and often frail populations are the main subject to amputation due to vascular disease and are rarely included in scientific studies that can evaluate different treatment options.^2^

Around the year 2000, Swedish authorities recognized the importance of using registers for quality control in healthcare. As a result, they began supporting the creation of new registers and established structured methods for financing and certifying these registers. This initiative has led to the development of over 100 healthcare registers.^3^ The national statistical agency, the Statistics Sweden official governmental statistics (SCB), covers a wide range of areas, such as demographics, economics, education, and labor for Sweden.^4^ This also includes the Swedish National Board of Health and Welfare (Socialstyrelsen) registers, responsible for regulating and supervising healthcare and social services across Sweden, ensuring quality and safety.^5^ It has administered several national registers since the start of inpatient data collection in 1964 and nationwide registration since 1987 to facilitate Swedish healthcare and social services analysis and development.^6^ The register's production and quality are monitored, and reports on the quality are published regularly.^7^ This and other Swedish registers are based on the Swedish personal identity number as an identifier. One of them is the Swedish National Inpatient Register (IPR), which includes a broad range of surgical operations (LLAs included) performed in Sweden since 1998.^8^ IPR uses the Swedish version of the Nordic Classification of Surgical Procedures codes (NCSP) for registration.^9^ It offers data on the number of surgeries performed, including LLAs divided into sex, age groups, and patient demographics.

In orthopedics, the Swedish Knee Arthroplasty Register was initiated in 1975 and was the first national register to monitor the outcome of a specific orthopedic surgical procedure. The SwedeAmp register,^10^ funded by the Swedish Association of Local Authorities and Regions, supports Sweden's municipalities and regions in delivering public services, including healthcare. The SwedeAmp register tracks data related to LLA, including limb loss due to vascular diseases, trauma, infections, and cancer. They focus specifically on rehabilitation with prostheses and collecting detailed data on:

- Amputation levels,
- Postoperative treatment,
- Prosthetic fitting, and
- Functional outcomes, published in a yearly report.^11^

The coverage of this register has improved since it started in 2011, from 16 hospitals covering patient data to include 36 hospitals (out of 67 that perform LLA) in 2023. Although not a nationwide register, it is currently the most extensive database related to LLA and outcomes globally and is published yearly.^12^ The SwedeAmp register has already shown sex differences concerning amputation level, diagnosis, and age, leading to the conclusion that women have worse preconditions for successful prosthetic mobility after LLA.^13^ However, the coverage rate has not been fully validated.

The Scottish Physiotherapy Amputee Research Group has made comparative register attempts^14^ reporting on LLAs in Scotland since 2015, and the latest report is from 2020–2021 (A survey of the lower limb amputee population in Scotland 2020 and 2021 public report). These can only be ordered through their website, and their data have not been externally evaluated. In the US, there is a plan by the Mayo Clinic to establish a limb loss and preservation registry to collect information to improve prevention, treatment, and rehabilitation efforts related to limb loss, but no data has been published.^15^

A recent Swedish study by Jarl et al. highlighted the need for a register to monitor LLAs. The study showed a national decline in LLA incidence (2008–2017) across most levels, except for partial foot amputations. Only 9 of 21 regions saw a combined decrease in LLAs, suggesting regional variations that merit further study, especially in below-knee vs. above-knee amputations.^16^ Another study by this group found higher LLA risks among elderly males with diabetes.^17^ One crucial example of how SwedeAmp data can be used is understanding the impact of the selection of amputation levels and how it affects rehabilitation outcomes. For instance, losing the knee joint, as seen in above-or through-knee amputations, significantly affects the function when a person is rehabilitated and uses a prosthesis.^18,19^ The knee joint is critical when it comes to mobility, balance, and life quality.^20^ Its absence requires patients to rely more heavily on the hip for movement, leading to slower walking speeds and increased energy expenditure.^21^

The AK/BK ratio can reveal surgical outcomes between clinics or regions. However, choosing the appropriate level depends on expertise, experience, and rehabilitation planning. Factors such as patient age and high rates of dysvascular conditions in certain areas can greatly impact both the chosen amputation level and the overall outcomes of the procedure.^17,22^ Trauma or sarcoma-related amputations are less common in developed countries and, therefore, contribute less to the overall amputation rates.^23^

Benchmarking is a relatively new tool for measuring and comparing outcomes, recently gaining attention in surgical practice.^24^ To the author's knowledge, it was first introduced in relation to lower limb amputation (LLA) in 1996.^25^ The future of benchmarking lies in developing national and international registries to establish standardized benchmarks. These databases ensure that data collection for specific procedures remains current, objective, standardized, and comprehensive. These systems allow healthcare providers to efficiently identify and monitor benchmarked and non-benchmarked interventions, creating accurate and relevant benchmarks. Naturally, this approach requires a commitment to participating in nationally approved data collection efforts.^26^

The next step for the SwedeAmp register's could be to establish benchmarking and incorporate known confounding factors, such as age, gender, diagnosis, comorbidities, postoperative treatments, prosthetic fitting technology, and access to rehabilitation, all of which affect rehabilitation outcomes after LLA.

High participation and population coverage in a register are critical for establishing a valid benchmark. The coverage rate of the SwedeAmp register for LLAs is unknown, while the IPR register, however, has a high coverage but only includes intervention codes and basic demographics. This study aimed to assess SwedeAmp's coverage against the more comprehensive nationwide IPR and proposed a benchmark based on frequent LLA levels. In subsequent studies, we plan to analyze the outcome data from the SwedeAmp register in more detail.

## METHODOLOGY

For this study, data from the IPR register was obtained from the period 2018–2023 from the Swedish National Board of Health and Welfare. The IPR provides open access data using the NCSP codes for registration of amputation levels (TF = NFQ19, KD = NGQ09, and TT = NGQ19), divided into the 21 regions of Sweden, five-year age groups and sex.^8^ Additionally, we applied to the IPR register for a list of all Hospitals performing these amputations. For comparison, we applied for data from the SwedeAmp register for the same period, utilizing their improved coverage rate and including the same parameters. Cooperating with the SwedeAmp register, hospitals use an online portal to report to the register in 6 different Forms.

Form 1 and 2 include all levels of LLA from partial toe amputation to hemipelvectomy, and Form 3–6 are solely focused on amputations at or proximal to TT amputation level^13^ using primarily ISO definitions.^27^ This data also included outcome data that will be used for analysis in later studies. For data regarding age and sex, the Official Statistics of Sweden (SCB) was used.^28^ The aim was to cross-reference data between the two registers to identify discrepancies. In our analysis, all surgical procedures were initial LLA performed on a limb. The person could, in theory, have been amputated before 1998 (before the start of the official IPR data collection), but the person occurs only once in our data sets. Due to inconsistent identification, certain hospital locations were grouped together to ensure database comparability. In the SwedeAmp database, one IPR hospital location (Halland Sjukhus) was split into two separate hospitals (Varberg and Halmstad).

### Statistics

***Incidence:*** Patient groups were divided into 5-year age intervals, except for those under 45 years old, who were grouped together due to the low incidence of LLA in this demographic in Sweden. The mean age-group data populations were calculated as the mean value for the population for each year of the study period (2018–2023).

This data was sourced from the SCB database.^28^ The mean amputation rates over the same period were obtained from the IPR database.^8^ The overall age-specific incidence rates for the initial LLA were thereafter calculated. The overall incidence per 100,000 person-years was calculated as the number of individuals who had undergone initial LLAs divided by the corresponding total population.

### Coverage rate

The SwedeAmp register's coverage rate was calculated using SwedeAmp data as the numerator and IPR data as the denominator.

### Benchmarking

To assess the ratio of amputation levels, we compared the average coverage rates from all 36 hospitals reporting to SwedeAmp. To control how the coverage rate changed with regard to amputation levels we focused on those hospitals that obtained 80%, 60%, and 40% coverage rates for comparison, using the IPR data as a reference. This conclusion is based on the observation that a higher coverage rate of over 80% would involve fewer patients and hospitals, and a lower percentage (less than 40%) would be the opposite scenario. Between 80% and 40% of this data formed the basis for establishing benchmark recommendations.

The result is presented stepwise:

- Overview of the IPR and SwedeAmp registered material with regional coverage;
- Demographics of SwedeAmp population by region;
- Regional comparison between IPR and SwedeAmp;
- Coverage rate by SwedeAmp hospitals
  - Comparison based on coverage rates of 80%, 60%, and 40%;
- Suggestion on a benchmark of amputation levels.

All statistical analyses were performed using R (R version 4.3.3, R Foundation for Statistical Computing, Vienna. Austria). Both the SwedeAmp and IPR (Inpatient Registry) databases were filtered and processed to include only relevant information for primary amputations. Various R packages were utilized for data manipulation, visualization, and statistical analysis. The tidyverse package was employed for general data manipulation and visualization tasks. The table1 package was used to create summary tables, while knitr and kableExtra were utilized to render tables in a publication-ready format. For color palettes, the RColorBrewer package was used. Spatial data manipulation and visualization were achieved using the swemaps2 and sf packages. Additionally, the stringr package was used for string manipulation.

Summary tables were created to describe the demographic and clinical characteristics of patients undergoing primary amputation in different regions.

To compare the distribution of demographic and clinical characteristics between the SwedeAmp and IPR databases, chi-square tests were performed. These tests assessed whether there were significant differences in the distributions between the two databases. Additionally, linear regression models were fitted to examine the relationship between coverage ratios and age, as well as coverage ratios and year. This analysis helped in understanding how coverage ratios varied with these factors.

Using the kable and kableExtra packages, summary tables were created to present data on amputation levels, gender distribution, and regional coverage. Spatial maps were generated to visualize the coverage of SwedeAmp by county and the population distribution by region. Line plots were created to show the trends in amputation type percentages based on hospital coverage rates and patient numbers.

This study was approved by the regional authorities KVB (nr. 152-24),^29^ and by the Swedish Ethical Review Authority, Dnr 2023-05222-01.

## RESULTS

### Overview of the registered material with regional coverage

According to IPR data, 67 hospitals across all 21 regions in Sweden performed a total of 10,788 initial LLAs at the TF, KD, and TT levels during the study period. 6,127 (56.8%) were male, 4,661 (43.2%) were women, averaging 85 amputations per region per year (range: 16–332). Sixteen of these hospitals reporting to IPR performed fewer than 10 LLAs over the six-year period. The SwedeAmp register collected data from 36 hospitals from 17 of the 21 regions on 5,246 LLAs during the same period. 2,994 (57.1%) were male, 2,252 (42.9%) were women, leaving 5,542 initial amputations unaccounted for in the SwedeAmp (**Table 1**). According to the IPR, to achieve full coverage from the 17 regions, SwedeAmp should have recorded 9,305 initial amputations (corresponding to 86.3% of all LLAs) during the study period.

**Table 1:** A systematic overview of the registered material.

Amputation Level	IPR		SwedeAmp*		Missing Data	
	n	%	n	%	n	%
**TF**	4,534	42.1	1,828	34.9	2,706	59.7
**KD**	800	7.5	438	8.3	362	45.3
**TT**	5,454	50.4	2,980	56.8	2,474	45.4
Sum *	10,788	100	5,246	100	5,542	**51.4

* 12 cases excluded due to missing information.

** Corresponding to the total % of missing data, not the sum of the column.

### Regional coverage

**Figure 1:**
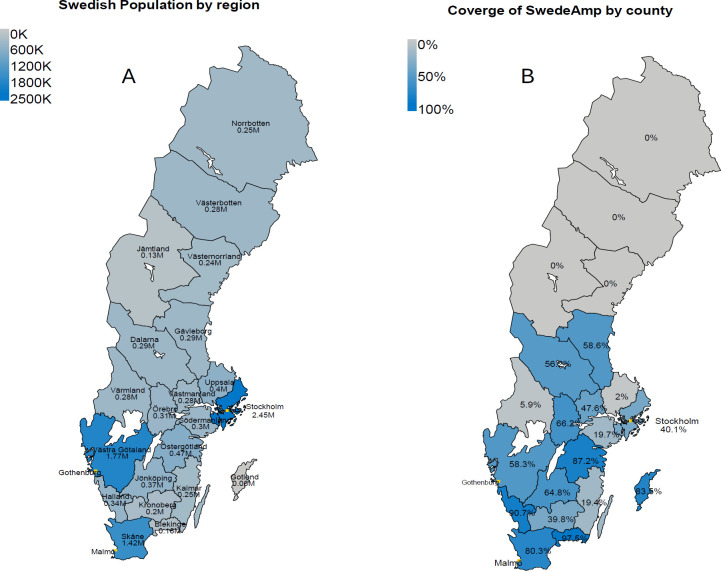
**A**: Geographical maps showing the Swedish population (by region). **B**: SwedeAmp coverage rate in % by regions compared to IPR.

A coverage map of Sweden's 21 regions shows that SwedeAmp primarily lacks data from the northern regions and two central regions that have not yet begun registration. (**Figure 1**).

The incidence of LLAs included in this data (TF, KD, and TT amputation only) was 16.5 amputations per 100,000 person-years. The highest incidence rate was found in men older than 80 and women older than 85. In SwedeAmp, individuals over the age of 85 were significantly underrepresented (**Table 2**).

**Table 2:** Characteristics of the study population stratified into sex and age groups compared with the data from IPR and SwedeAmp (with different coverage rates).

			Standard Population	Incidence	IPR	SwedeAmp (100%)	SwedeAmp Hospitals With >80% Coverage
Sex	Age-Group	Average Annual Amputations in Sweden (2018–2023)	Average Annual Population in Sweden (2018–2023)	Amputation per 100.000 Person-Years	% of Total Amputees	% of Total Amputees	% of Total Amputees
Men	0–44	22.5	2,967,692	0.8	1.3%	1.3%	1.4%
Men	45–49	9.3	390,037	2.4	0.5%	0.7%	0.7%
Men	50–54	21.7	396,103	5.5	1.2%	1.6%	1.6%
Men	55–59	40.5	382,253	10.6	2.3%	2.5%	2.2%
Men	60–64	53.7	334,992	16.0	3.0%	3.5%	3.2%
Men	65–69	90.7	313,105	29.0	5.1%	5.5%	5.3%
Men	70–74	162.5	307,995	52.8	9.2%	9.5%	9.7%
Men	75–79	203.7	258,017	78.9	11.5%	11.1%	11.5%
Men	80–84	184.3	152,914	120.5	10.4%	9.9%	9.7%
Men	85+*	219.3	114,181	192.1	12.3%	11.3%	10.6%
**Sum**		**1008.2**	**5,617,289**	**17.9**	**56.8%**	**57.0%**	**56.0%**
Women	0–44	13.8	2,789,703	0.5	0.8%	0.8%	0.8%
Women	45–49	8.0	324,070	2.5	0.5%	0.6%	0.8%
Women	50–54	9.7	330,276	2.9	0.5%	0.7%	0.7%
Women	55–59	18.5	319,789	5.8	1.0%	1.1%	0.9%
Women	60–64	25.0	285,328	8.8	1.4%	1.7%	1.9%
Women	65–69	47.7	273,753	17.4	2.7%	3.2%	3.4%
Women	70–74	89.7	277,640	32.3	5.1%	5.0%	5.4%
Women	75–79	122.8	240,766	51.0	6.9%	7.4%	7.3%
Women	80–84	136.5	159,630	85.5	7.7%	7.6%	8.2%
Women	85+*	296.0	170,714	173.4	16.7%	15.0%	14.5%
**Sum**		**767.7**	**5,171,668**	**14.8**	**43.2%**	**43.0%**	**44.0%**
**Men + Women**		**1,775.9**	**10,788,957**	**16.5**	**100%**	**100%**	**100%**

* People over the age of 85 are significantly underrepresented.

### Demographics of SwedeAmp population by region

The registration revealed consistency and disparity from regions reporting into SwedeAmp (**Table 3**). When three regions (Uppsala, Värmlands, and Västerbottens region) were excluded due to low registrations, the rate of TF amputation varied between 8.8% and 56.1%, the rate of KD amputation showed variation between 4.0% and 36.0%, and the rate of TT amputation varied between 31.8% and 86.8%. However, the ratio of amputation side was similar, and the median age ranged between 73 and 79 years in these regions. Men were more represented in all regions, with a ratio of 1.38/1 (range = 51.9% to 72.1%). In the IPR database, the ratio was similar, men (n = 6,127) and women (n = 4661), resulting in a 1.31/1 ratio.

**Table 3:** Demographics of the SwedeAmp population by region (n = 17). The Uppsala, Värmland, and Västerbotten regions are excluded from further statistical analysis due to their low registration rates.

	Dalarnas Region	Jönköpings Region	Östergötlands Region	Skåne Region	Stockholms Region	Västra Götalands Region
	(N=181)	(N=294)	(N=394)	(N=1069)	(N=798)	(N=1135)
**Amputation Level**						
TF	38 (21.0%)	103 (35.0%)	125 (31.7%)	385 (36.0%)	180 (22.6%)	527 (46.4%)
KD	31 (17.1%)	19 (6.5%)	142 (36.0%)	48 (4.5%)	32 (4.0%)	51 (4.5%)
TT	112 (61.9%)	172 (58.5%)	127 (32.2%)	636 (59.5%)	586 (73.4%)	557 (49.1%)
**Amputation Side**						
Left	83 (45.9%)	148 (50.3%)	189 (48.0%)	527 (49.3%)	374 (46.9%)	568 (50.0%)
Right	98 (54.1%)	146 (49.7%)	205 (52.0%)	542 (50.7%)	424 (53.1%)	567 (50.0%)
**Age at Amputation**						
Mean (SD)	77.9 (10.8)	76.3 (13.1)	74.9 (13.1)	75.9 (12.1)	77.3 (12.7)	76.1 (12.9)
Median [Min, Max]	79 [26, 101]	78 [24, 101]	77 [22, 99]	78 [20, 101]	79 [22, 101]	78 [21, 100]
**Gender**						
Women	72 (39.8%)	138 (46.9%)	183 (46.4%)	447 (41.8%)	334 (41.9%)	525 (46.3%)
Men	109 (60.2%)	156 (53.1%)	211 (53.6%)	622 (58.2%)	464 (58.1%)	610 (53.7%)
**Year**						
2018	34 (18.8%)	69 (23.5%)	58 (14.7%)	185 (17.3%)	115 (14.4%)	134 (11.8%)
2019	26 (14.4%)	63 (21.4%)	64 (16.2%)	185 (17.3%)	152 (19.0%)	217 (19.1%)
2020	29 (16.0%)	41 (13.9%)	69 (17.5%)	145 (13.6%)	130 (16.3%)	158 (13.9%)
2021	29 (16.0%)	27 (9.2%)	55 (14.0%)	161 (15.1%)	83 (10.4%)	183 (16.1%)
2022	37 (20.4%)	50 (17.0%)	77 (19.5%)	198 (18.5%)	122 (15.3%)	239 (21.1%)
2023	26 (14.4%)	44 (15.0%)	71 (18.0%)	195 (18.2%)	196 (24.6%)	204 (18.0%)
	**Blekinge Region**	**Gävleborgs Region**	**Gotlands Region**	**Hallands Region**	**Örebro Region**	**Västmanlands Region**
	**(N=195)**	**(N=177)**	**(N=81)**	**(N=380)**	**(N=231)**	**(N=107)**
**Amputation Level**						
TF	103 (52.8%)	49 (27.7%)	34 (42.0%)	106 (27.9%)	62 (26.8%)	60 (56.1%)
KD	11 (5.6%)	26 (14.7%)	12 (14.8%)	27 (7.1%)	16 (6.9%)	13 (12.1%)
TT	81 (41.5%)	102 (57.6%)	35 (43.2%)	247 (65.0%)	153 (66.2%)	34 (31.8%)
**Amputation Side**						
Left	99 (50.8%)	91 (51.4%)	41 (50.6%)	192 (50.5%)	109 (47.2%)	57 (53.3%)
Right	96 (49.2%)	86 (48.6%)	40 (49.4%)	188 (49.5%)	122 (52.8%)	50 (46.7%)
**Age at Amputation**						
Mean (SD)	78.0 (11.7)	73.9 (14.1)	77.2 (11.1)	78.4 (11.1)	76.4 (10.5)	77.0 (12.7)
Median [Min, Max]	79 [21, 99]	75 [22, 98]	79 [45, 93]	79 [30, 101]	78 [41, 98]	79 [21, 98]
**Gender**						
Women	74 (37.9%)	76 (42.9%)	39 (48.1%)	155 (40.8%)	102 (44.2%)	39 (36.4%)
Men	121 (62.1%)	101 (57.1%)	42 (51.9%)	225 (59.2%)	129 (55.8%)	68 (63.6%)
**Year**						
2018	27 (13.8%)	9 (5.1%)	15 (18.5%)	45 (11.8%)	47 (20.3%)	12 (11.2%)
2019	34 (17.4%)	10 (5.6%)	16 (19.8%)	58 (15.3%)	44 (19.0%)	14 (13.1%)
2020	37 (19.0%)	42 (23.7%)	9 (11.1%)	62 (16.3%)	48 (20.8%)	22 (20.6%)
2021	30 (15.4%)	38 (21.5%)	7 (8.6%)	78 (20.5%)	28 (12.1%)	18 (16.8%)
2022	30 (15.4%)	31 (17.5%)	14 (17.3%)	56 (14.7%)	33 (14.3%)	21 (19.6%)
2023	37 (19.0%)	47 (26.6%)	20 (24.7%)	81 (21.3%)	31 (13.4%)	20 (18.7%)
	**Kalmar Region**	**Kronobergs Region**	**Södermanlands Region**	**Uppsala Region**	**Värmlands Region**	
	**(N=47)**	**(N=68)**	**(N=64)**	**(N=9)**	**(N=16)**	
**Amputation Level**						
TF	17 (36.2%)	6 (8.8%)	27 (42.2%)	1 (11.1%)	5 (31.3%)	
KD	4 (8.5%)	3 (4.4%)	3 (4.7%)	0 (0%)	0 (0%)	
TT	26 (55.3%)	59 (86.8%)	34 (53.1%)	8 (88.9%)	11 (68.8%)	
**Amputation Side**						
Left	25 (53.2%)	33 (48.5%)	33 (51.6%)	5 (55.6%)	6 (37.5%)	
Right	22 (46.8%)	35 (51.5%)	31 (48.4%)	4 (44.4%)	10 (62.5%)	
**Age at Amputation**						
Mean (SD)	70.3 (16.4)	75.0 (10.4)	75.8 (11.3)	65.9 (14.9)	66.9 (8.59)	
Median [Min, Max]	73 [22, 94]	77 [41, 92]	77 [50, 92]	65 [44, 89]	69 [47, 81]	
**Gender**						
Women	19 (40.4%)	19 (27.9%)	25 (39.1%)	0 (0%)	5 (31.3%)	
Men	28 (59.6%)	49 (72.1%)	39 (60.9%)	9 (100%)	11 (68.8%)	
**Year**						
2018	10 (21.3%)	9 (13.2%)	0 (0.0%)	3 (33.3%)	1 (6.3%)	
2019	6 (12.8%)	12 (17.6%)	0 (0.0%)	1 (11.1%)	0 (0.0%)	
2020	20 (42.6%)	8 (11.8%)	3 (4.7%)	1 (11.1%)	9 (56.3%)	
2021	3 (6.4%)	12 (17.6%)	2 (3.1%)	2 (22.2%)	2 (12.5%)	
2022	2 (4.3%)	9 (13.2%)	20 (31.3%)	2 (22.2%)	0 (0.0%)	
2023	6 (12.8%)	18 (26.5%)	39 (60.9%)	0 (0.0%)	4 (25.0%)	

### Regional comparison

The data highlights a significant disparity in clinical practice regarding the selection of amputation levels. Five regions showed a higher ratio of AK amputation (Östergötlands, Västra Götalands, Blekinge, Gotlands and Västmanlands). The difference was most transparent in Östergötlands and Hallands regions in the IPR database and the SwedeAmp register, with similar coverage rates.

#### IPR Database Data:

- Östergötlands region: AK/BK ratio of 1.72/1 (63.3% above-knee vs. 36.7% below-knee).
- Hallands region: AK/BK ratio of 0.69/1 (40.9% above-knee vs. 59.1% below-knee).

While the IPR data showed Östergötland favoring above-knee amputations and Halland favoring below-knee amputations, the ratios differ slightly from those reported in SwedeAmp.

#### SwedeAmp Register Data:

- Östergötlands region: AK/BK ratio of 2.10/1 (67.7% above-knee vs. 32.2% below-knee).
- Hallands region: AK/BK ratio of 0.54/1 (35.0% above-knee vs. 65.0% below-knee).

Still, Östergötland performs a far higher proportion of above-knee amputations than Halland region.

### Comparison using ≥40% coverage rate

Data from SwedeAmp, from 13 regions in Sweden that obtained at least ~40% coverage of the IPR register, was used for further analysis (**Table 4**). In total, these regions in SwedeAmp registered 5,110 amputations (61.8% coverage), 2,901 TT (56.7%), 1,778 TF (34.8%), and 431 KD (8.4%), of which 2,907 (56,9%) were male and 2,203 (43,1%) women. The IPR registered during the same period 8,262 amputations (4,266 TT (51.6%), 3,280 TF (39.7%), and 716 KD (8.7%), of which 4,736 (57.3%) were male and 3,526 (42.7%) women.

**Table 4:** List of regions included in the coverage analysis (regions with at least 40% coverage in the SwedeAmp register are specified and included in the further analysis on amputation levels).

Region	Subjects in IPR Database	Subjects in SwedeAmp Database	SwedeAmp Coverage (%)	Included in Analysis
Blekinge län	200	195	97.5	Yes
Dalarnas län	322	181	56.2	Yes
Gotlands län	97	81	83.5	Yes
Gävleborgs län	302	177	58.6	Yes
Hallands län	419	380	90.7	Yes
Jämtlands län	165	0	0.0	No
Jönköpings län	454	294	64.8	Yes
Kalmar län	242	47	19.4	No
Kronobergs län	171	68	39.8	Yes
Norrbottens län	474	0	0.0	No
Skåne län	1,332	1,069	80.3	Yes
Stockholms län	1,992	798	40.1	Yes
Södermanlands län	325	64	19.7	No
Uppsala län	443	9	2.0	No
Värmlands län	271	16	5.9	No
Västerbottens län	275	0	0.0	No
Västernorrlands län	331	0	0.0	No
Västmanlands län	225	107	47.6	Yes
Västra Götalands län	1,947	1,135	58.3	Yes
Örebro län	349	231	66.2	Yes
Östergötlands län	452	394	87.2	Yes
**Sum**	**10,788**	**5,246**		(5,110)*

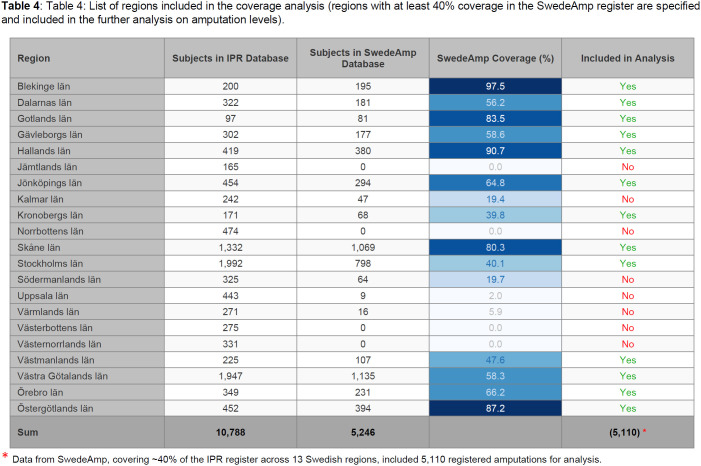

* Data from SwedeAmp, covering ~40% of the IPR register across 13 Swedish regions, included 5,110 registered amputations for analysis.

**Figure 2:**
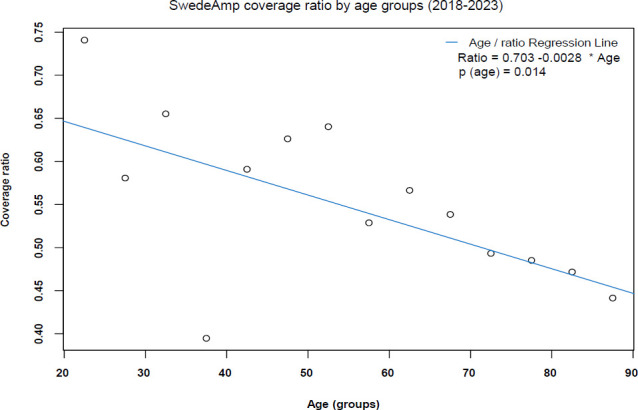
SwedeAmp coverage ratio by age groups (2018–2023).

**Figure 3:**
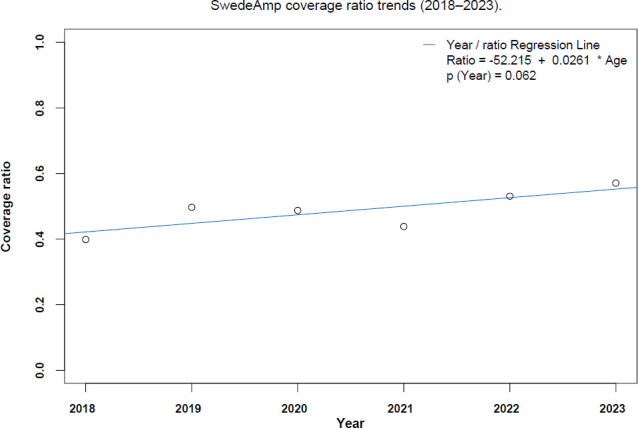
SwedeAmp coverage ratio trends (2018–2023).

When analyzing the coverage ratio by age groups, the older the patients were, the higher the risk of not being included in the data from SwedeAmp (**Figure 2**). The hospital coverage ratio by year showed that despite the impact of the pandemic in 2021, coverage still rises yearly (**Figure 3**).

### SwedeAmp coverage rate by hospitals

Thirty-six out of the 67 hospitals were included in the SwedeAmp database. Two hospitals in the SwedeAmp register were reported as one hospital in the IPR database (**Table 5**).

**Table 5:** List of hospitals performing LLAs and represented in both IPR and SwedeAmp data (n=36).

Hospital	Patients in IPR	Patients in SwedeAmp	Coverage Rate (%)
Akademiska sjukhuset Uppsala	441	9	2,0
Blekingesjukhuset	200	195	97,5
Capio S:t Görans sjukhus	258	181	70,2
Centrallasarettet Växjö	143	68	47,6
Centralsjukhuset Karlstad	238	16	6,7
Centralsjukhuset Kristianstad	212	182	85,8
Danderyds sjukhus	405	253	62,5
Falu lasarett	234	181	77,4
Hallands sjukhus*	419	380	90,7
Helsingborgs lasarett	290	195	67,2
Hudiksvalls sjukhus	101	2	2,0
Hässleholms sjukhus	2	1	50,0
Höglandsjukhuset Eksjö	157	84	53,5
Karolinska Univ sjukhuset (Huddinge + Solna)	461	170	36,9
Kungälvs sjukhus	32	12	37,5
Lasarettet i Motala	78	37	47,4
Länssjukhuset Ryhov Jönköping	214	209	97,7
Länssjukhuset i Kalmar	157	34	21,7
Mälarsjukhuset i Eskilstuna	229	60	26,2
Norrtälje sjukhus	107	9	8,4
Nyköpings lasarett	93	4	4,3
Sahlgrenska Univ sjukhus (Göteborg + Mölndal)	704	525	74,6
Sjukhuset i Gävle	200	175	87,5
Skånes Univ.sjukhus (Malmö + Lund)	723	676	93,5
Södersjukhuset	669	141	21,1
Södertälje Sjukhus	91	44	48,4
Södra Älvsborgs sjukhus Borås	357	267	74,8
Uddevalla NÄL	411	331	80,5
Univ sjukhuset Linköping	192	186	96,9
Univ sjukhuset Örebro	339	231	68,1
Visby lasarett	97	81	83,5
Vrinnevisjukhuset i Norrköping	182	171	94,0
Värnamo sjukhus	83	1	1,2
Västerviks sjukhus	85	13	15,3
Västmanlands sjukhus Västerås	225	107	47,6
Ystad Lasarett	91	15	16,5
**Sum**	**8,920**	**5,246**	

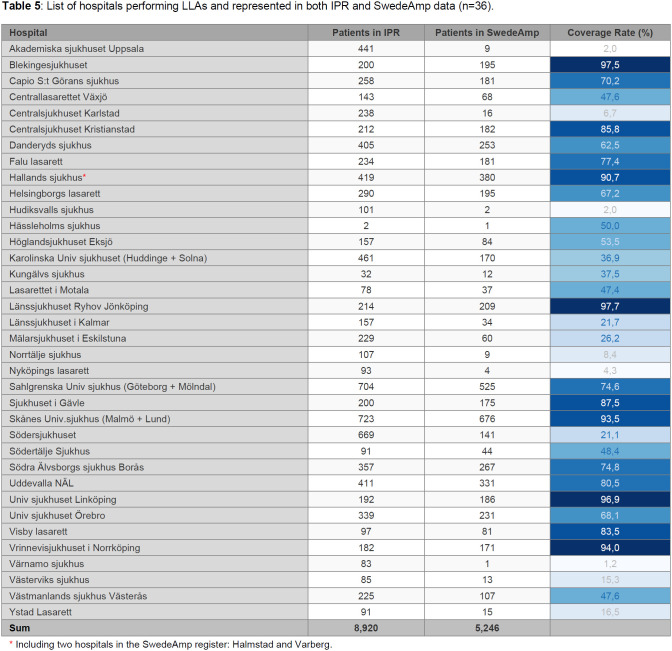

* Including two hospitals in the SwedeAmp register: Halmstad and Varberg.

The SwedeAmp register collected 5246 cases during the study period from 36 hospitals. On average, 24 patients went through LLA per year and hospital (range = 0.2–112.7). However, six hospitals performed less than ten LLAs (26 LLAs in total). Excluding these hospitals, the average yearly number will rise to 29 persons per year and hospital (n = 30 hospitals, 5220 patients, range = 2.0–112.7) (**Table 5**).

Six hospitals reported over 90% of amputations: 1,817 out of 1,936 (93.9% coverage rate). Four more hospitals reported between 80% to 89% of amputations: 769 out of 921 (83.5%). Fourteen hospitals reported 40%–79% amputation: 2,159 out of 3,303 (65.1% coverage rate). In total, 24 hospitals obtained a coverage rate of ≥40% (**Table 5**).

### Suggestion on a benchmark of amputation levels

We calculated the ratio using hospital reports to SwedeAmp with a coverage rate of more than 60% for one or more years (N = 36). The data includes a total of 4,419 amputations. With a suggested minimum coverage rate of ≥60%, the proposed benchmark ratios were ≤34.3% for transfemoral (TF) amputations, ≤8.4% for knee disarticulations (KD, where the knee joint is lost), and ≥55.3% for transtibial (TT) amputations. Minimal ratio changes were observed when using coverage rates of 80% or 40% (**Table 6**).

**Table 6:** SwedeAmp amputations by level in all, 80%, 60%, and 40% hospital coverage rates.

Amputation Level	Count in All	Ratio in All	Count in Hospitals	Ratio in Hospitals	Count in Hospitals	Ratio in Hospitals	Count in Hospitals	Ratio in Hospitals
	All Hospitals (N = 36)		≥80% (N = 10)		≥60% (N = 17)		≥40% (N = 24)	
**TF**	1,828	34.9%	932	36.0%	1,603	**36.3%**	1,699	35.7%
**KD**	438	8.4%	267	10.3%	373	**8.4%**	415	8.72%
**TT**	2,980	56.8%	1,387	53.6%	2,443	**55.3%**	2,646	55.6%
**Total**	**5,246**	**100%**	**2,586**	**100%**	**4,419**	**100%**	**4,760**	**100%**

## DISCUSSION

The SwedeAmp register covered 48.6% of all lower limb amputations (LLAs) performed during the study period, representing 17 out of 21 regions in Sweden and 5,246 cases. The four non-participating regions were in northern Sweden, three of which are the largest by area. While these regions have lower population densities, this alone does not fully explain their absence from the register. In total, eight regions either do not report to the register or have less than 20% coverage. Data from 36 hospitals (53.7%) out of the 67 performing LLAs across the 21 regions were included in the registry between 2018 and 2023.

This study's incidence report using the IPR database was lower than previous studies (17) as it only represents the TT, KD, and TF amputation levels. However, we also looked at the trend from 2008 to 2023 using the IPR database, and gladly, the incidence is declining, even after 2018.^8^

The reported data showed a notable trend of older patients being underrepresented. This may be due to the focus of healthcare professionals involved in the rehabilitation phase, who tend to concentrate on the outcomes of their patients rather than on those who are not enrolled in intensive rehabilitation program or who pass away shortly after surgery.

Interestingly, out of the 13 regions that reported more than 20% coverage rate, one region (Östergötland) reported a 36.0% rate of KD amputations, while four regions reported between 12.1 % and 17.1%, and the remaining eight regions were all below 8.5% of all LLAs. A notable difference was found related to the AK/BK ratio, where two regions, Västmanland (56.1%) and Blekinge (52.8%), reported that more than half of all amputations were performed on the TF level. Additionally, three more regions reported a higher AK/BK ratio (when the KD level is included), while seven regions reported a reverse AK/BK ratio. These regions have a substantial number of LLAs and high coverage, with median age and range similar to other regions with different ratios. No significant age-related differences were observed between the cohorts. These disparities warrant further exploration and may justify using amputation levels as a benchmark for comparison.

However, there is an indication that patients not reported to SwedeAmp were from the oldest age group in the population. The question is not whether the 49% of missing data confounds the material represented in the SwedeAmp reports; it's more related to how much it affects. The effect can be addressed differently. We have demonstrated that although data is missing, the level of amputations in hospitals showed similar disparities when using 80% coverage compared with 40% coverage.

The average of 29 initial amputations per year and hospital, ranging from 2.0 to 112.7 patients, is noteworthy. According to the IPR register, sixteen hospitals performed fewer than ten amputations over six years, including six in the SwedeAmp register. Due to these low numbers, these hospitals were excluded from further statistical analysis.

Further investigation is needed to determine whether the volume of procedures at these hospitals impacts patient outcomes compared to hospitals that perform LLAs more frequently, or if this discrepancy could be due to incorrect registrations. These findings revealed the question of whether LLAs should be considered a specialized are of focus to improve the outcome in the future.

The SwedeAmp initiative, based on hospitals' voluntary participation, focuses on patient-centered care by tracking clinical outcomes, patient satisfaction, quality of life, and functional mobility.

Although no guidelines on LLA in Sweden are available, benchmarking against best practices or, as here, using the outcome data from SwedeAmp could help reduce variability and ensure that patients receive the most appropriate level of amputation.

Addressing these differences allows healthcare providers to provide more consistent and equitable treatment for patients undergoing LLA nationwide. However, a key challenge for SwedeAmp, as highlighted in this study, is ensuring consistent data reporting from all healthcare providers. Reporting gaps can hinder nationwide conclusions and obscure certain trends.

International comparisons of SwedeAmp data are primarily limited to Scottish reports,^14^ as no other comparable registries exist. Notable discrepancies arise when comparing the initial level of amputation between these registries. In Scotland, 60% of amputations are TT, 1% are KD, and 39% are TF amputations,^14^ compared to 56.8%, 8.3%, and 34.9% in SwedeAmp, respectively. Additionally, the average age of LLA in Scotland is 67 years, which is ten years younger than the average age in Sweden.^16^

A deeper analysis of the patient characteristics and clinical decision-making processes in different geographic regions worldwide and within Sweden could provide insights into the factors driving the current discrepancies. This might involve looking closer at age, gender, comorbidities, and the availability of limb-salvage interventions. SwedeAmp data has the potential to become a valuable resource for exploring how social determinants of health influence post-amputation recovery and rehabilitation outcomes.

Benchmarking levels can be justified in several ways.^26^ In this study, we selected those hospitals with adequate registration coverage when comparing the IPR with SwedeAmp. Another approach would be to choose the ‘best’ hospital, defined by high volume, a low AK/BK ratio, and low mortality rates for amputations, and use that as the benchmark. However, the same result would have been obtained since high-volume centers have similar ratios.

A third approach involves selecting a ‘perfect’ candidate for amputation and benchmarking based on those cases. However, this approach is less realistic for LLAs due to age variations and the high comorbidity burden in this population. A potential limitation of this study is the discrepancy between the data provided by the administration managing the IPR register (Swedish National Board of Health and Welfare) and the online data from the same database, which showed a difference of 168 cases (1.6%). According to the data provider, this discrepancy may be due to instances where the same patient undergoes amputations at different hospitals, with each hospital independently registering the procedure. Another limitation is the uncertainty regarding the impact of population ethnicity and regional comorbidity rates, both of which are known to influence outcomes following LLA. Additionally, outcomes can be affected by factors such as socioeconomic status, healthcare access, and genetic predispositions, which are not fully accounted for in the current SwedeAmp register.^30^

These findings highlighted the need for broader SwedeAmp participation to achieve comprehensive, reliable nationwide data on LLAs. Expanded participation strengthens SwedeAmp's value as a research resource, allowing for deeper insights into procedural efficacy, regional disparities, and patient care improvements. This will ultimately promote higher standards in both local and national healthcare.

## CONCLUSION

The SwedeAmp register encompasses over half of all amputations documented in the IPR database. This study underscored the variations observed in both registries, particularly concerning lower limb amputations (LLAs) in terms of age, amputation levels, and geographic distribution. Notable differences in the above-knee (AK) to below-knee (BK) amputation ratios were identified across various regions. Additionally, our findings indicated inconsistencies in age group representation within the SwedeAmp data. Based on the insights from the SwedeAmp data, we have proposed benchmark recommendations regarding amputation levels of ≤36.3% for TF, ≤8.4% for KD, and ≥55.3% for TT amputations.

## DECLARATION OF CONFLICTING INTERESTS

**Anton G. Johannesson** is an employee of Össur Clinics, which provides services to prosthetic and orthotics clinics. **Reynir Scheving, Karolin Lindgren Westlund**, and **Thor Fridriksson** are all employed by Össur Iceland ehf, Medical Office in Reykjavik, Iceland.

## AUTHORS CONTRIBUTION

- **Anton G. Johannesson**: Conceptualization; Study oversight; Data analysis; Writing original; Review and editing.
- **Reynir Scheving**: Conceptualization; Study oversight; Data analysis; Review and editing.
- **Karolin Lindgren Westlund**: Conceptualization; Study oversight; Review and editing.
- **Thor Fridriksson**: Conceptualization; Study oversight; Review and editing.

All authors reviewed the manuscript and approved the final version.

## SOURCES OF SUPPORT

No external support was obtained for this project.

## DEFINITIONS/ABBREVIATIONS

**AK/BK Ratio**: The Ratio of Above-Knee (AK) Amputations (TF+KD) to Below-Knee (BK) Amputations (TT) Within a Given Population.
**Initial Limb Amputation**: The First LLA Surgery on a Person's Limb.
**IPR**: The Swedish National Inpatient Register.
**NCSP**: Nordic Classification of Surgical Procedures (Swedish version).
**SCB**: Statistics Sweden (official Governmental Statistics).
**SwedeAmp**: The Amputation and Prosthetics Registry for the Lower Limb.
**LLA**: Lower Limb Amputation.
**KD**: Knee Disarticulation.
**TF**: Transfemoral.
**TT**: Transtibial.
